# The effects of enhancing angiotensin converting enzyme in myelomonocytes on ameliorating Alzheimer’s-related disease and preserving cognition

**DOI:** 10.3389/fphys.2023.1179315

**Published:** 2023-06-23

**Authors:** Ron Danziger, Dieu-Trang Fuchs, Yosef Koronyo, Altan Rentsendorj, Julia Sheyn, Eric Y. Hayden, David B. Teplow, Keith L. Black, Sebastien Fuchs, Kenneth E. Bernstein, Maya Koronyo-Hamaoui

**Affiliations:** ^1^ Department of Neurosurgery, Maxine Dunitz Neurosurgical Institute, Cedars-Sinai Medical center, Los Angeles, CA, United States; ^2^ Department of Neurology, Cedars-Sinai Medical Center, Los Angeles, CA, United States; ^3^ Department of Neurology, David Geffen School of Medicine at UCLA, Mary S. Easton Center for Alzheimer’s Disease Research at UCLA, Brain Research Institute, Molecular Biology Institute, University of California, Los Angeles, CA, United States; ^4^ College of Osteopathic Medicine of the Pacific, Western University of Health Sciences, Pomona, CA, United States; ^5^ Department of Biomedical Sciences, Cedars-Sinai Medical Center, Los Angeles, CA, United States; ^6^ Department of Pathology and Laboratory Medicine, Cedars-Sinai Medical Center, Los Angeles, CA, United States

**Keywords:** microglia, monocytes, macrophages, innate immune cells, degrading peptidase, pro-inflammatory cytokines, neurodegenerative diseases, neuroscience

## Abstract

This review examines the role of angiotensin-converting enzyme (ACE) in the context of Alzheimer’s disease (AD) and its potential therapeutic value. ACE is known to degrade the neurotoxic 42-residue long alloform of amyloid β-protein (Aβ_42_), a peptide strongly associated with AD. Previous studies in mice, demonstrated that targeted overexpression of ACE in CD115^+^ myelomonocytic cells (ACE10 models) improved their immune responses to effectively reduce viral and bacterial infection, tumor growth, and atherosclerotic plaque. We further demonstrated that introducing ACE10 myelomonocytes (microglia and peripheral monocytes) into the double transgenic APP_SWE_/PS1_ΔE9_ murine model of AD (AD^+^ mice), diminished neuropathology and enhanced the cognitive functions. These beneficial effects were dependent on ACE catalytic activity and vanished when ACE was pharmacologically blocked. Moreover, we revealed that the therapeutic effects in AD^+^ mice can be achieved by enhancing ACE expression in bone marrow (BM)-derived CD115^+^ monocytes alone, without targeting central nervous system (CNS) resident microglia. Following blood enrichment with CD115^+^ ACE10-monocytes versus wild-type (WT) monocytes, AD^+^ mice had reduced cerebral vascular and parenchymal Aβ burden, limited microgliosis and astrogliosis, as well as improved synaptic and cognitive preservation. CD115^+^ ACE10-versus WT-monocyte-derived macrophages (Mo/MΦ) were recruited in higher numbers to the brains of AD^+^ mice, homing to Aβ plaque lesions and exhibiting a highly Aβ-phagocytic and anti-inflammatory phenotype (reduced TNFα/iNOS and increased MMP-9/IGF-1). Moreover, BM-derived ACE10-Mo/MΦ cultures had enhanced capability to phagocytose Aβ_42_ fibrils, prion-rod-like, and soluble oligomeric forms that was associated with elongated cell morphology and expression of surface scavenger receptors (i.e., CD36, Scara-1). This review explores the emerging evidence behind the role of ACE in AD, the neuroprotective properties of monocytes overexpressing ACE and the therapeutic potential for exploiting this natural mechanism for ameliorating AD pathogenesis.

## 1 Introduction

A hallmark of early pathological changes in Alzheimer’s Disease (AD) is accumulation of amyloid β-protein (Aβ) in the brain and retina (Selkoe, 2001; Hardy and Selkoe, 2002; Selkoe, 2008; Shankar et al., 2008; Koronyo-Hamaoui et al., 2011; La Morgia et al., 2016; Koronyo et al., 2017; Shi et al., 2020; Koronyo et al., 2023). The amyloidogenic 42-residue long alloform (Aβ_42_) is particularly important due to its ability to rapidly assemble into highly synaptotoxic soluble oligomers as well as aggregate into insoluble fibrils and form plaques (Li et al., 2020). In particular, the Aβ_42_ oligomers (oAβ_42_) exert downstream effects on cell signaling pathways that ultimately result in functional-structural synaptic and neuronal abnormalities (Shankar et al., 2007; Li et al., 2020). One potential mechanism for this is via the binding of oligomeric Aβ to the cellular prion protein receptor (PrP^C^), which forms a complex with the metabotropic glutamate receptor 5 (mGluR5) at the cell surface. This subsequently results in synergistic co-activation of Pyk2 (Protein rich tyrosine kinase 2) and Fyn (Fyn proto-oncogene, Src Family Tyrosine Kinase). Fyn then phosphorylates tau which results in neuronal dysfunction (Brody and Strittmatter, 2018). Another proposed mechanism implicates the N-methyl-D-aspartate (NMDAR) type glutamate receptor, which at low frequency synaptic stimulation enhances long term depression (LTD) via a calcineurin/cofilin dependent pathway that leads to spine retraction. In experimental models, soluble Aβ enhances this pathway and ultimately leads to dendritic spine loss (Shankar et al., 2007). It has also been shown that accumulation of Aβ_42_ may lead to reactive oxygen species (ROS) generation (Cenini et al., 2010). One proposed mechanism is that Aβ_42_ activates caspase signaling pathways in cerebral cortical neurons and leads to upregulation of pathways involved in mitochondrial fission. This also leads to the reduction of mitochondrial membrane potentials resulting in increased ROS generation with subsequent activation of mitophagy and eventual neuronal apoptosis (Han et al., 2017). These cellular pathways, together with other pathological processes such as chronic neuroinflammation and vascular deficiencies are hypothesized to reduce synaptic plasticity and result in the debilitating cognitive deficits that characterize this devastating illness (Walsh et al., 2002; Shankar and Walsh, 2009; Tu et al., 2014; Umeda et al., 2015; Yu et al., 2019). Given the injurious role that soluble Aβ_42_ oligomers play in AD pathophysiology, there has been considerable effort to target and remove them as a strategy to prevent disease progression (Viola and Klein, 2015; Honig et al., 2018; Alexander et al., 2021; Mintun et al., 2021).

Aβ peptides are catabolized by peptidases that can convert these peptides to shorter, less pathologically active forms (Mukherjee et al., 2000; Kanemitsu et al., 2003; Hemming and Selkoe, 2005; Yin et al., 2006). One of which is angiotensin-converting enzyme (ACE), a zinc dipeptide carboxypeptidase containing two proteolytically active homologous regions, designated the N- and C-domains (Wei et al., 1991; Bernstein et al., 2013). ACE is expressed ubiquitously by endothelium as well as cortical neurons and is well known for its role in conversion of angiotensin II to angiotensin I and maintenance of hemodynamic stability (Kehoe et al., 2009). ACE can also be expressed by circulating monocytes, tissue macrophages and microglia (Bernstein et al., 2014; Cao et al., 2020a). On monocytes, most ACE is membrane bound but some can be released into the extracellular space by protease activity at the cell surface (Wei et al., 1991; Bernstein et al., 2013). There now exists evidence that ACE may play a pivotal role in cleavage of Aβ by promoting degradation of naturally occurring Aβ_42_ and Aβ_40_ peptides (Hemming and Selkoe, 2005) and the conversion of Aβ_42_ into shorter, less pathogenic alloforms (Zou et al., 2007), and thus has a protective role against the accumulation of synaptotoxic and neurotoxic oligomeric and fibril forms. More recent studies have demonstrated that macrophages that express higher levels of ACE degrade extracellular Aβ_42_ faster and more efficiently than WT macrophages (Koronyo-Hamaoui et al., 2020).

In addition, there is emerging evidence that certain microglial subtypes, and moreover peripherally-derived monocytes and macrophages (Mo/MΦ), once believed to have purely detrimental effects in AD pathophysiology, now may play a neuroprotective role, home to sites of amyloid deposition and are crucial in modulating chronic inflammation and facilitating Aβ phagocytosis and degradation (Butovsky et al., 2006; Simard et al., 2006; Butovsky et al., 2007; Deane et al., 2009; Koronyo-Hamaoui et al., 2009; Marques et al., 2009; El Khoury, 2010; Lebson et al., 2010; Michaud et al., 2013; Koronyo et al., 2015; Keren-Shaul et al., 2017; Zuroff et al., 2017; Rentsendorj et al., 2018; Doustar et al., 2020; Koronyo-Hamaoui et al., 2020; Dvir-Szternfeld et al., 2022). Enhanced expression of ACE in CD115^+^ myelomonocytic cells (ACE10 or ACE^10/10^ model) improve their ability to mediate an effective immune response to remove viral and bacterial infections, cancer growth, and atherosclerotic plaques (Shen et al., 2007; Okwan-Duodu et al., 2010). Importantly, it has been demonstrated that targeted overexpression of ACE in CD115^+^ myelomonocytic cells (i.e., monocytes, macrophages, microglia) in the double-transgenic APP_SWE_/PS1_ΔE9_ murine models of AD (AD^+^ mice), increased cerebral Aβ clearance, curbed neuroinflammation, and strikingly, preserved memory and learning functions (Bernstein et al., 2014). These effects were dependent on ACE catalytic activity, which was lost when ACE was pharmacologically blocked by the ACE inhibitor ramipril. Notably, adoptive transfer of young WT, and moreover, ACE^10/10^ bone marrow (BM)-derived CD115^+^ monocytes to the peripheral blood of AD^+^ mice, enhanced their recruitment to cerebral Aβ lesion sites, reduced parenchymal and vascular amyloidosis, and restored synaptic integrity (Koronyo-Hamaoui et al., 2020). These ACE^10/10^ monocytes exhibited neuroprotective properties with improved phagocytosis of Aβ_42_ fibrils and oligomers as well as reduced production of pro-inflammatory cytokines such as tumor necrosis factor α (TNFα) and inducible nitric oxide synthase (iNOS), with elevated production of matrix metallopeptidase 9 (MMP-9) and neurotrophic Insulin-like growth factor 1 (IGF-1). Consequently, these AD^+^ mice had a marked reduction in brain amyloid plaque area and soluble Aβ_42_ levels, which was reflected in improved cognitive testing and preserved spatial memory and learning capacity.

The mechanism by which ACE expression regulates the secretion of these cytokines is not well understood but there is emerging evidence that ACE overexpression aids in metabolic reprograming associated with the polarization of not only monocytes, but all myeloid cells. ACE enhances the metabolic state of these cells by increasing ATP, Krebs cycle intermediates, and electron transport chain proteins (Cao et al., 2020b). ACE also has an important function in antigen presenting cells (APCs) as it functions as a peptidase involved in peptide trimming and enhances major histocompatibility complex (MHC) class 1 epitope presentation. Furthermore, ACE changes MHC class II presentation of peptide epitopes via unclear mechanisms hence driving CD4 differentiation towards different phenotypes which in turn alters the cytokine profile present in the inflammatory milieu (Bernstein et al., 2018).

This review explores the emerging evidence behind the role of ACE in AD, the neuroprotective properties of monocytes overexpressing ACE and the therapeutic potential for exploiting this natural mechanism for amyloid clearance.

## 2 The role of ACE in AD

There now exists extensive histological, biochemical and genetic evidence for the role of ACE in AD. In an analysis of parenchymal tissue by quantitative immunofluorescence, frontal cortex samples from patients with AD without cerebral amyloid angiopathy (CAA) were compared to those with more severe CAA as well as controls (Miners et al., 2008). In AD patients, neuronal and perivascular ACE expression was increased and correlated with parenchymal Aβ levels in a statistically significant way. Perivascular ACE levels were elevated in patients with CAA in particular. This suggests that ACE may be upregulated in cases of increased Aβ deposition as its activity confers an important role in amyloid degradation. It also raises the possibility that this natural mechanism of raising ACE in response to Aβ is not sufficient to clear vascular Aβ in AD patients and requires further enhancement. In another *postmortem* analysis, ACE levels but not activity was reduced in the cerebrospinal fluid (CSF) of AD patients and correlated with increased Braak staging, a marker of topographic progression of AD (Miners et al., 2009). These findings are thought-provoking as increasing Braak staging denotes neurofibrillary tangle involvement progressing through the transentorhinal, limbic and then neocortical regions (Braak and Braak, 1991). Increased Braak staging has been correlated with the degree and severity of memory decline (Grober et al., 1999). As AD progresses through the brain, ACE activity may increase as a protective mechanism to promote amyloid clearance. This was shown by CSF studies in living patients that demonstrated increased ACE activity in patients with mild cognitive impairment (MCI) and AD when compared to healthy controls (He et al., 2006; Jochemsen et al., 2014).

While these studies show a correlation between ACE levels and disease severity, they do not solidify a causal relationship between ACE activity and amyloid clearance. There are indeed alternate hypotheses that suggest the renin angiotensin aldosterone system (RAAS) plays a key role in neuroinflammation and cerebrovascular attenuation and may itself be implicated in the pathogenesis of AD (Mateos et al., 2011; Kehoe et al., 2019). This could imply that elevated ACE in the CSF is merely a byproduct of CNS RAAS upregulation in AD rather than ACE itself being selectively upregulated to clear amyloidogenic protein. However, Genome Wide Association Studies (GWAS) indicate ACE as risk factor for AD (Meng et al., 2006; Marioni et al., 2019; Xin et al., 2021) and GWAS data on ACE polymorphisms show a correlation between low ACE activity and increased AD severity (Meng et al., 2006; Marioni et al., 2019). In one study of living patients with subjective memory complaints or diagnosed AD, ACE protein levels as well as Aβ_42_ levels were measured in the CSF. Both ACE protein levels and activity were lower in the CSF of patients with diagnosed AD. Lower CSF ACE protein levels were associated with lower CSF Aβ_42_ levels which suggests more accumulation of Aβ in the brain parenchyma (Jochemsen et al., 2014). Again, from this study, it can be postulated that ACE levels and activity are directly correlated with amyloid clearance.

Given the discussion so far, ACE is functionally able to degrade pathogenic amyloid protein and human tissue studies demonstrate that ACE may serve a protective role in combatting cerebral Aβ accumulation. It stands to reason that if this mechanism is deranged by decreasing ACE activity in the brain, the amyloidogenic proteins may be able to freely accumulate. Perhaps a genetic aberration may result in this derangement. The ACE gene is located on chromosome 17q23. Several studies have attempted to elucidate ACE gene polymorphisms that result in increased susceptibility to AD. One large recent meta-analysis of 65 studies involving 82 cohorts of differing ethnic composition and comprising of 47,000 genotyped cases and controls implicated the rs1799752 polymorphisms with susceptibility for development of AD (Xin et al., 2021). This is the insertion/deletion (I/D) variant of 287-bp intron 16 and is the most common polymorphism of the ACE gene. Carriers of the D allele have higher levels of plasma ACE compared to I homozygotes and almost 50% of variability in plasma ACE levels are dependent on this polymorphism in the human population. When analyzing association of each allele with risk of AD, the I allele was shown to confer an increased susceptibility to AD compared to the D allele. However, an ethnic difference was also noted with a significant association only found in North European populations but not in East Asian populations or those of South European descent. Other meta-analysis studies have implicated other insertion/deletion variants of intron 16 that may reduce plasma levels of ACE and contribute to pathogenesis of disease (Lehmann et al., 2005; Bertram et al., 2007). For example, GWAS analysis has revealed that the single nucleotide combination of rs4343 and rs4351, two adjacent polymorphisms in the DCP1/ACE gene that are linked to the same Insertion variant, may result in up to a 45-fold increased risk of developing AD (Meng et al., 2006; Marioni et al., 2019).

## 3 The role of microglia and monocytes in AD pathology

It is crucial to consider more broadly the role of microglia and monocytes in AD pathophysiology before launching into a discussion of the important role of monocytes overexpressing ACE. While traditionally conceptualized as a purely neurodegenerative disease, recent advances have demonstrated the neuroinflammatory basis of AD, which predominantly includes activated brain-resident microglia and reactive astrocytes but also infiltrating cells from the peripheral immune system and the subsequent pro-inflammatory milieu and neuronal damage that ensues (Prinz and Priller, 2017; Rossi et al., 2021). Systemic inflammation may contribute to the neuroinflammation implicated in the neurodegenerative cascade of AD (Zhang et al., 2013). Additionally, microglia may be primed by the toxic environment in the AD brain to then differentiate towards a more maladaptive inflammatory phenotype (Cunningham, 2013). Microglia exert these detrimental effects by aggregating around Aβ plaques and secreting high levels of pro-inflammatory cytokines such as TNFα and IL-6 which result in significant neurotoxicity (Xu et al., 2016).

While monocytes and microglia can have the aforementioned deleterious effects in AD pathophysiology, there is emerging evidence for the neuroprotective and anti-inflammatory roles that subpopulations of these pleomorphic cells may have (Morgan et al., 2005; Koronyo-Hamaoui et al., 2009; Lebson et al., 2010; Michaud et al., 2013; Koronyo et al., 2015; Rentsendorj et al., 2018; Li et al., 2020). Recent *in vivo* imaging studies have demonstrated the capacity for circulating peripheral immune cells to directly clear cerebral Aβ (Michaud et al., 2013). Using two photon microscopy in murine models of AD it was demonstrated that patrolling Ly6C^lo^ monocytes crawl on the luminal walls of Aβ-positive veins, phagocytose Aβ and then circulate back into the bloodstream. This finding is particularly important as CAA is critical in AD pathogenesis and is correlated with worsening cognition (Charidimou et al., 2017). Perivascular and parenchymal Aβ exist in a state of equilibrium. Hence reduction of perivascular Aβ may result in an efflux of parenchymal Aβ to restore this equilibrium. This could potentially result in a decreased burden of parenchymal Aβ and contribute to reduced neurodegeneration (Deane et al., 2009; Marques et al., 2009; Pimentel-Coelho and Rivest, 2012).

In a 2015 study the ways in which peripheral monocytes can be harnessed for Aβ clearance was explored (Koronyo et al., 2015). It was shown that weekly immunization of AD^+^ mice with glatiramer acetate (GA) resulted in enhanced recruitment of peripheral monocytes to Aβ plaques, decreased burden (45%–50% reduction) of these plaques and retained cognition. In this experiment CD115^+^- monocytes were injected once monthly into the peripheral blood of AD^+^ mice with or without weekly GA injection and compared to a cohort treated with GA injection alone as well as an age matched control. Every treatment group exhibited a substantial decrease in cognitive deficits assessed by Barnes Maze testing, increased synaptic perseveration, and marked reduction in cerebral Aβ levels and astrogliosis. Fluorescently labelled peripherally engrafted monocytes were localized around cerebral amyloid plaques and engulfed Aβ. This effect was enhanced in the GA immunized mice and was associated with increased IL-10 and MMP-9 levels. Further *in vitro* studies using cultured BM demonstrated that GA treated macrophages could phagocytose fibrillar Aβ_42_ (fAβ_42_) and exhibited increased expression of scavenger receptors CD36, SCARA1 and MMP9, all of which has been shown to facilitate Aβ phagocytosis (Coraci et al., 2002; Yin et al., 2006; Frenkel et al., 2013). These results were significant as they indicate that peripheral monocytes, even without ACE overexpression, play a critical role in phagocytosis and degradation of pathogenic Aβ that may result in synaptic preservation, reduced astrogliosis and cognitive improvement.

In another study, the specific mechanisms by which BM-derived macrophages protected synapses against pathogenic Aβ was explored (Li et al., 2020). Firstly, it was found that oAβ_42_, more-so than fAβ_42_ inhibited neuritic arborization, induced loss of excitatory VGluT1/PSD95 synapses and caused functional alterations in neurons. When co-cultured with fAβ_42_ and oAβ_42_, BM-derived macrophages were able to remove these pathogenic species. Fibrils were cleared via CD36/EEA1^+^ early endosomal proteolysis while oligomers were removed via extracellular/MMP-9 enzymatic degradation. *In vivo* analysis revealed enhanced entorhinal cortex and hippocampal preservation of excitatory VGluT1/PSD95 synapses in GA immunized and CD115^+^-monocyte-grafted AD^+^ mice compared to AD^+^ mice control. A 2022 study using genetic fate-mapping demonstrated that 6% of amyloid plaque associated macrophages are derived from circulating hematopoietic originating monocytes rather than brain native microglia which are yolk sac derived (Yan et al., 2022). Additionally peripheral monocytes enriched the choroid plexus, meninges and perivascular spaces more so in aged AD mice than WT control. Splenectomy reduced circulating monocytes, thereby reducing amyloid plaque associated macrophages of a peripheral origin and resulted in increased amyloid plaque burden. Thus, despite making up a small amount of plaque associated myelomonocytes, those of a peripheral origin clearly play a significant role in tempering AD pathogenesis.

Microglia too, can have a protective effect in AD. A recent study using single cell transcriptomic techniques uncovered a rare microglia sub-population termed disease associated microglia (DAM), involved in restricting cerebral Aβ plaque formation. Histologically these DAM cells were frequently located near Aβ plaques in both human and mice brains. Immunohistochemical staining of both mice and human AD brains show DAMs with intracellular phagocytosed Aβ, suggesting their ability to absorb Aβ peptides and a protective role in AD. In normal physiology, DAM activation is tightly controlled via checkpoint mediated inhibition. Regulation occurs via a 2-step process. In the first step a Trem2-independent process occurs which involves reduced expression of homeostatic microglia checkpoint genes such as Cx3cr1 and P2ry12/P2ry12 and upregulation of B2m, Apoe and Tyrobp. This intermediate DAM is then further activated by a Trem2-dependent process involving regulation of phagocytic and lipid metabolism genes such as CSt7 and Lpl. It is feasible that the inhibition of some of these microglia specific inhibitory checkpoints (such as Cx3cr1) which in turn disinhibits DAM activation could prove to be an important therapeutic target in the future (Keren-Shaul et al., 2017).

A parallel study identified a similar subpopulation of microglia, termed neurodegeneration-associated microglia (MGnD) controlled by TREM2-APOE pathway and post-transcriptionally regulated by microRNA(miR)-155 in the proximity of plaques, implying their detrimental role in neurodegeneration (Krasemann et al., 2017). Ultimately further studies are needed into these DAM or MGnD microglia, particularly when it comes to the inciting triggers in the AD brain microenvironment that skew microglia towards either of these unique phenotypes.

## 4 ACE overexpression in microglia and monocytes

As discussed, ACE is an important zinc peptidase capable of degrading Aβ. In addition, microglia and monocytes play a crucial inflammatory role in AD and themselves are capable of clearing Aβ aggregates via mechanisms involving cellular uptake and enzymatic degradation. It is apparent that even in the absence of ACE overexpression, monocytes play an important role in mitigating AD pathology. Prior studies have demonstrated that the adoptive transfer of BM-derived CD115^+^ monocyte subsets (WT for ACE expression) from young donor mice (8–10 weeks old) into 10-month-old AD^+^ mice leads to diminished neuropathology and preserved cognitive function (Koronyo et al., 2015).

In humans and mice, ACE is expressed on monocytes and is naturally upregulated in multiple disease processes. In particular, macrophages and myeloid derived giant cells express abundant ACE in many granulomatous diseases (for example,: sarcoidosis, leprosy, tuberculosis, histoplasmosis and Wegener’s granulomatosis) (Lieberman, 1975; Williams and Williams, 1983; Brice et al., 1995; Baudin, 2002; Bernstein et al., 2018). Hence it was long postulated that ACE may improve the antibacterial effectiveness of macrophages. In mice, ACE expression in neutrophils, macrophages and dendritic cells rapidly increases following *Staphylococcus aureus* or *Listeria* infection (Okwan-Duodu et al., 2010). ACE is also upregulated in non-infectious disease processes such as atherosclerotic disease. Macrophages identified in both early and late-stage human atherosclerotic material were found to produce abundant ACE (Diet et al., 1996; Ohishi et al., 1997). It is now known that ACE overexpression not only improves bacterial defenses but actually enhances the adaptive and innate immune response via a diverse array of mechanisms. This includes not only improved antibacterial defenses but also antigen processing, enhanced antitumor responses and improved antibody production (Bernstein et al., 2013; Bernstein et al., 2018; Koronyo-Hamaoui et al., 2014; Cao et al., 2023).

**FIGURE 1:**
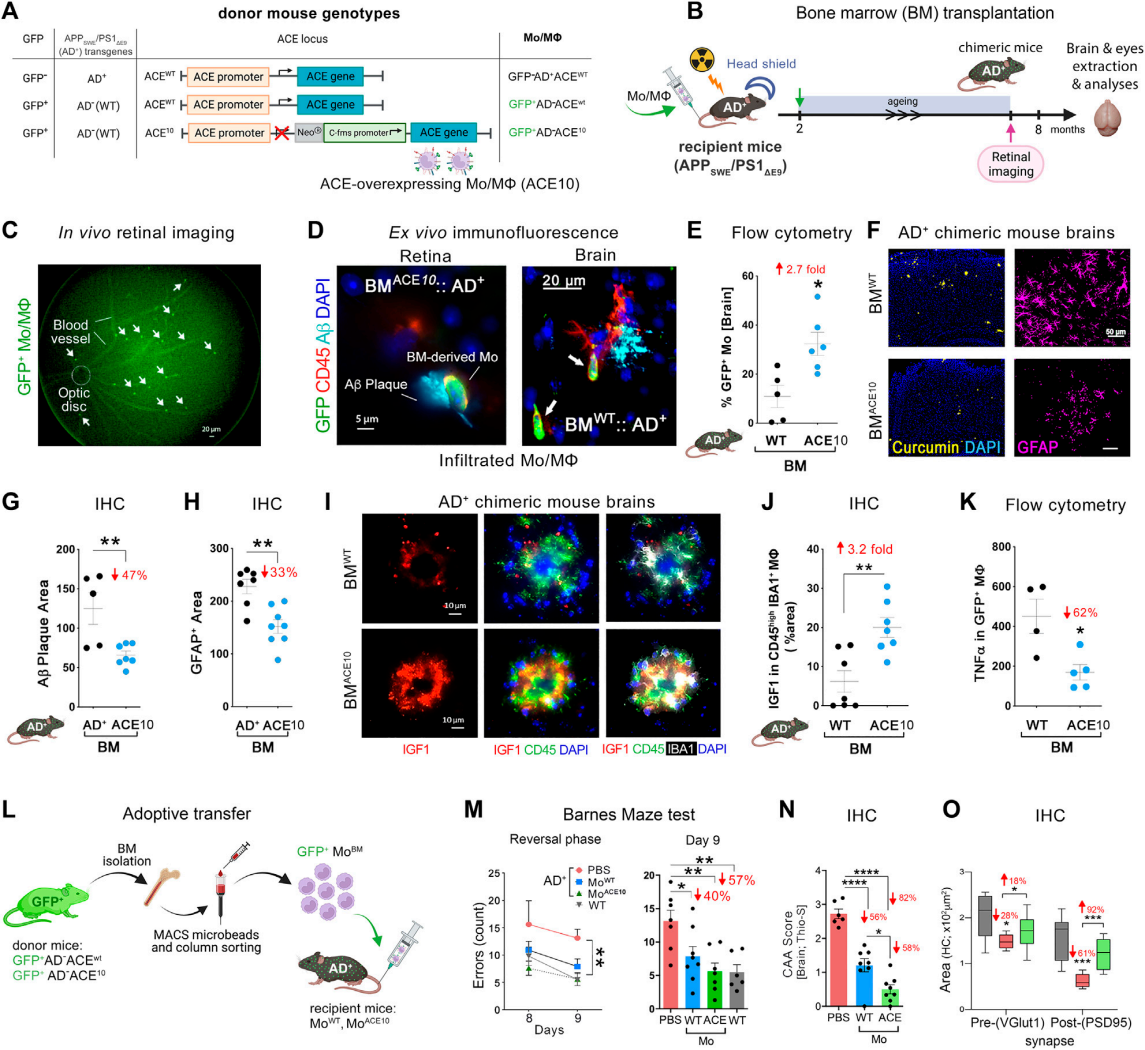
Neuroprotection by ACE-overexpressing monocytes and macrophages in APP_SWE_/PS1_ΔE9_ (AD^+^) mice. **(A)** Description of the genetic composition for the 3 groups of bone marrow (BM) donor mice at the GFP, APP_SWE_/PS1_ΔE9_ (AD^+^), and ACE loci: GFP^−^AD^+^ACE^WT^, GFP^+^AD^−^ACE^WT^, or GFP^+^AD^−^ACE^10^. In the ACE10 mouse models, ACE expression is controlled by the c-fms (CD115) promoter that substituted the endogenous ACE promoter. This results in the over expression of ACE in monocytes and macrophages (Mo/MΦ). **(B)** BM transplantation experiments: 2-month-old double transgenic AD^+^ recipient mice underwent irradiation with head shielding to prevent brain irradiation and inflammation. Their BM was then reconstituted by an intravenous injection of 5 million BM cells from one of the three types of donors. This BM transplantation generated BM^AD*+*^, BM^WT^, and BM^ACE10^ chimeric AD^+^ mice. These mice underwent retinal imaging at the age of 7 months, followed by perfusion and harvest of eyes and brains for analysis. **(C)** A representative live fluorescence image of a BM-chimeric AD^+^ mouse retina, demonstrating the feasibility to noninvasively detect *in vivo* infiltrating GFP^+^ Mo/MΦ in the retina. [**(D)**, left] Fluorescence micrograph of a BM^ACE10^::AD^+^ chimeric mouse retina (previously *in vivo* imaged) showing the homing of GFP^+^/CD45^hi^ ACE^10^ Mo/MΦ to the site of retinal 4G8^+^-Aβ deposit. Scale bar = 5 μm. [**(D)**, right] Infiltrating Mo/MΦ (GFP^+^/CD45^hi^; arrows) at the 6E10^+^-Aβ plaque site are also detected in the BM^WT^::AD^+^ chimeric mouse brain. Scale bar = 20 μm. **(E)** Quantitative flow cytometry analysis of cerebral infiltrating GFP^+^-Mo/MΦ in BM^ACE10^ versus BM^WT^ chimeric AD^+^ mice. **(F)** Micrographs of curcumin^+^-Aβ plaques (yellow) or GFAP^+^ astrocytes (pink) in the cortices of BM^WT^ versus BM^ACE10^ AD^+^ chimeric mice. Scale bar = 50 μm. **(G)** Quantitative immunohistochemical (IHC) analysis of 6E10^+^-Aβ plaque area in the cortex of BM^ACE10^ versus BM^AD+^ AD^+^-chimeric mice. **(H)** Quantitative IHC analysis of cortical GFAP^+^ astrocytosis area in AD^+^-chimeric mice. **(I)** Representative fluorescent micrographs of cortical brain regions stained against IGF-1 (red) combined with myeloid cell markers, IBA1 (white) and CD45 (green), and nuclei (DAPI, blue) in BM^WT^ versus BM^ACE10^ AD^+^-chimeric mice. Scale bars = 10 μm. Brain-infiltrating CD45^hi^ IBA1^+^ ACE10-versus WT-Mo/MΦ highly expressed IGF-1 in AD^+^-chimeric mice. **(J)**. Quantitative IHC analysis of % IGF-1^+^ area colocalized with Mo/MΦ in BM^WT^ versus BM^ACE10^ AD^+^-chimeric mice. **(K)** Quantitative flow cytometry analysis of TNFα expression levels in infiltrating ACE10-versus WT-MΦ (GFP^+^ CD11b^+^ CD45^hi^ Ly6C^int-hi^ F4/80^hi^) in chimeric AD^+^ mice. **(L)** An additional study involved adoptive transfer of 5-6 million ACE10-or WT-CD115^+^ monocytes (Mo^BM^) from young donor mice into the peripheral blood of 8-month-old AD^+^ mice. These inflammatory monocytes were isolated from the BM of GFP^+^ donor mice and enriched in the tube using the CD115-positive MACS column sorting procedure. ACE10- (Mo^ACE10^) or WT- (Mo^WT^) BM were then injected into the tail vein of recipient AD^+^ mice. At the age of 11 months, mice underwent behavioral testing. **(M)** Barnes maze cognitive test: average errors count during the reversal phase, days 8 and 9. Statistics: (left) two-way ANOVA and Bonferroni’s post-test; (right) Day 9 memory test by one-way ANOVA and Bonferroni’s post test. **(N)** Severity of vascular Aβ deposits is defined as CAA score assessed with thioflavin-S (Thio-S^+^) staining in AD^+^ mice brain. **(O)** Quantitative IHC analysis of hippocampal pre-(VGluT1^+^) and post-(PSD95^+^) synaptic areas in Mo^ACE10^-treated mice compared to age- and gender-matched PBS-injected control AD^+^ and naïve WT mice. Illustration in panels A, B and L were created with Biorender.com, as well as images and graphs were used as original or modified from (Koronyo-Hamaoui et al., 2020) by permission of Oxford University Press.

Further studies have explored the unique immunomodulatory functions of myelomonocytes overexpressing ACE in the context of AD and discovered that these cells possess uniquely enhanced neuroprotective properties and an enhanced capacity to clear Aβ (Bernstein et al., 2014). This phenomenon has been examined using the ACE10 mouse model. In this model, gene targeting is used to inactivate the intrinsic ACE promoter and subsequently place ACE expression under the c-fms promoter. ACE is then overexpressed in the myelomonocytic lineage. Homozygous ACE^10/10^ mice lack ACE expression by any tissue that does not recognize the c-fms promoter including endothelial cells. While the heterozygous ACE^10/WT^ mice retain endogenous ACE expression and have increased ACE expression by myelomonocytes (Figure 1A). In situations where the immune response is challenged directly by tumor or bacterial infection these mice demonstrated an increased immune response with substantially enhanced monocyte recruitment to sites of inflammation (Shen et al., 2007; Okwan-Duodu et al., 2010).

In an important 2014 study (Bernstein et al., 2014) ACE^10/10^ mice were crossed with double-transgenic AD^+^ mice. This is an established AD mouse model whereby these mice overproduce amyloidogenic Aβ_42_ peptides and show progressive memory deficits. AD^+^ mice with WT ACE alleles (AD^+^ACE^WT/WT^), heterozygous for the ACE10 allele (AD^+^ACE^10/WT^) and homozygous for this allele (AD^+^ACE^10/10^), as well as WT mice (AD^–^ACE^WT/WT^) were compared. Histological quantification of hippocampal and cortical Aβ plaques demonstrated a marked reduction in plaque burden in AD^+^ mice with the ACE10 allele(s), Plaque area was significantly reduced in AD^+^ACE^10/WT^ mice versus AD^+^ACE^WT/WT^ mice. This reduction was greater in AD^+^ACE^10/10^ mice, which showed a reduced plaque burden of 69%–79% compared with AD^+^ACE^WT/WT^ mice (*p* < 0.0001). Histological analysis also revealed substantially less perivascular Aβ deposition for AD^+^ACE^10/WT^ mice compared to either AD^+^ACE^WT/WT^ or AD^+^ACE^10/10^ mice. Interestingly AD^+^ACE^10/10^ mice lack ACE expression in vascular endothelium. This highlights the critical importance of both intra parenchymal and endothelial ACE in clearance of Aβ. Soluble amyloidogenic Aβ_42_ was also reduced in mice overexpressing ACE in the myelomonocytic lineage (Bernstein et al., 2014).

Astrogliosis is an important marker of AD pathophysiology that may lead to neuronal damage and the devastating cognitive effects of the disease (Wyss-Coray and Mucke, 2002). Interestingly, AD^+^ACE^10/WT^ and AD^+^ACE^10/10^ mice also saw a significant reduction of 45%–75% in astrogliosis compared to AD^+^ACE^WT/WT^ mice at 7 months. This was maintained at 13 months with a 50% reduction in astrogliosis in AD^+^ACE^10/10^ mice compared to AD^+^ACE^WT/WT^ mice. This marked decrease in astrogliosis was reflected in cognitive testing. Barnes maze testing in 11- and 12-month-old mice with at least one ACE10 allele showed intact maze solving capabilities not statistically different from non-transgenic AD^–^ACE^WT/WT^ control mice. Thus, the presence of even a single ACE10 allele allows for reduction of amyloidogenic Aβ_42_, reduction in vascular and parenchymal Aβ burden, reduced astrogliosis and microgliosis and thus preserved cognition.

ACE enriched monocytes appears to play a pivotal role in tempering AD pathogenesis. In another 2020 study, the selective contribution of monocytes, but not microglia, overexpressing ACE was further explored (Koronyo-Hamaoui et al., 2020). Two approaches were applied in AD^+^ mice recipients. In one approach, BM from GFP^+^ACE^10/10^ donor mice was transplanted into AD^+^ mice whereby the recipients were lead head shielded from BM suppressive radiation to prevent extraneous infiltration of peripheral immune cells induced by the irradiation (Figures 1B–K). In the other approach adoptive transfer of CD115^+^- monocytes from GFP^+^ACE^10/10^ young donor mice into the peripheral blood of recipient AD^+^ mice took place; this procedure enriched by ∼5-folds the population of circulating CD115^+^- monocytes in the blood of the recipient mice (Figures 1L–O). These *in vivo* studies revealed the reduction in parenchymal and vascular Aβ deposits by 47%–82%, soluble Aβ_42_ levels by 31%, and GFAP^+^ astrogliosis cell number and area by 33% in AD^+^ mice that had received BM transplantation or adoptive transfer of GFP^+^ACE^10/10^ marrow compared to GFP^+^ACE^WT/WT^ marrow control (Figures 1G,H). As in the BM transplantation experiment described above (Koronyo-Hamaoui et al., 2020), not only is there clearly less plaque burden but so too a reduction in amyloidogenic Aβ_42_ and a significant reduction in astrogliosis. These ACE overexpressing monocytes took on a neuroprotective phenotype and surrounded brain and retinal amyloid plaques. These cells expressed 3.2-fold higher insulin-like growth factor-1 (IGF-1), a protein with anti-inflammatory functions implicated in neuroprotection in AD (George et al., 2017; Arjunan et al., 2023), while expressing 60% less TNFα, implicated in harmful neurotoxic effects in AD (Chang et al., 2017; Liddelow et al., 2017) (Figures 1J,K). This was true even in subsequent *in vitro* studies with ACE^10/10^ bone marrow-derived macrophages that exhibited distinct Aβ-phagocytic, anti-inflammatory, neuroprotective immune profiles with high IGF-1, and scavenger receptors (i.e., CD36, Scara-1 and MMP-9) as well as low iNOS and an elongated cell morphology even after direct exposure to Aβ_42_ soluble oligomers (Koronyo-Hamaoui et al., 2020).

**FIGURE 2:**
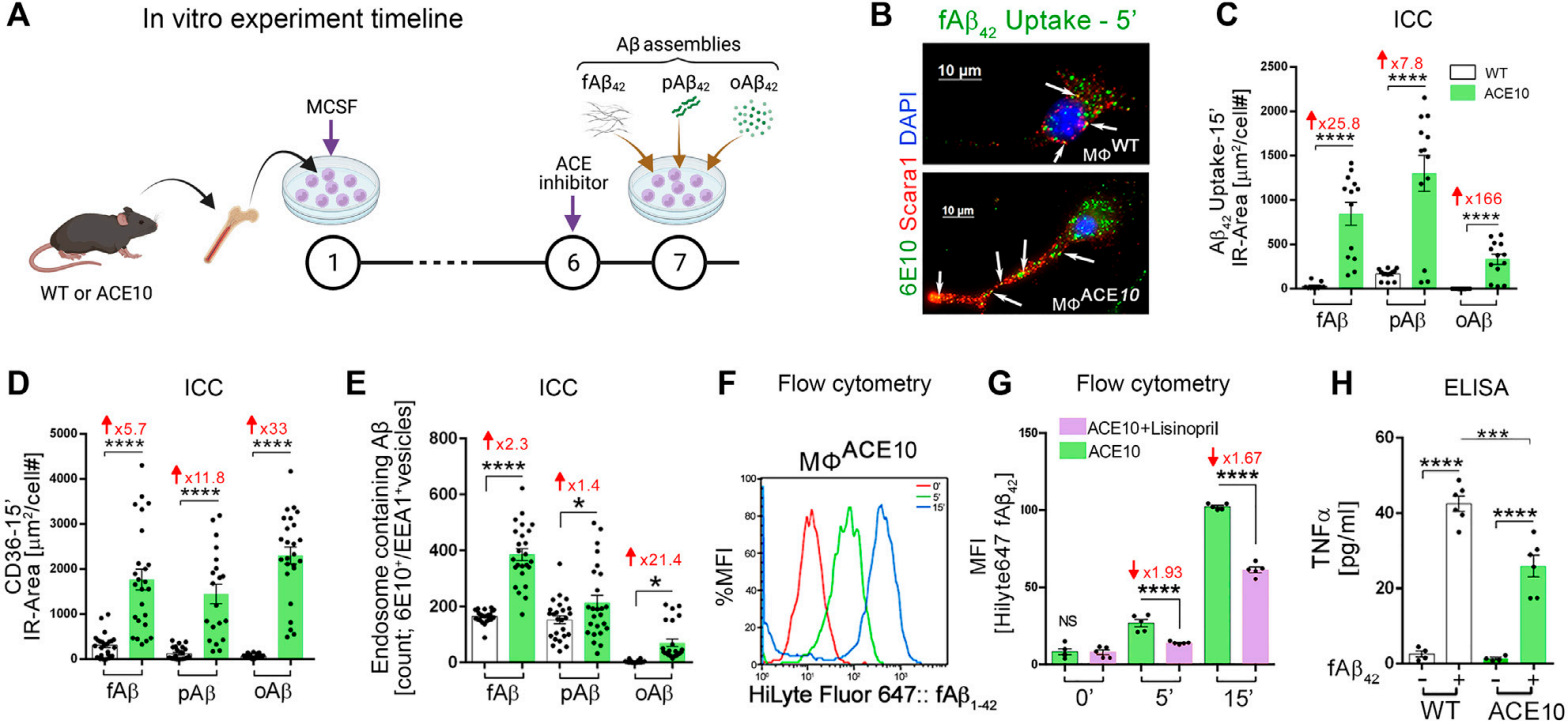
Enhanced clearance of Aβ_42_ assemblies by ACE-overexpressing macrophages. **(A)** *In vitro* experimental procedure: BM was isolated from young WT and ACE10 mice (8–12 weeks old) and cultured for 6 days with macrophage colony stimulating factor (MCSF)-enriched media, generating primary macrophage cultures (MΦ^WT^ and MΦ^ACE10^, respectively). On Day 7, MΦ were either unstimulated (Un) or stimulated by one of the 3 Aβ_42_ forms: fibrils (fAβ_42_), prion rod-like structures (pAβ_42_), or cross-linked oligomers (oAβ_42_). MΦ^ACE10^ were analyzed and compared to control MΦ^WT^ for cell surface Aβ_42_ binding and receptor expression as well as uptake of Aβ_42_ species at incremental time points from 5 to 15 min (incubated at 37°C). Additional experimental groups were pretreated at day 6 with the ACE inhibitor lisinopril, blocking its two catalytic domains. **(B)** Representative fluorescence micrograph of MΦ^WT^ and MΦ^ACE10^ cells that were incubated for 5 min with 250 nM fibril Aβ_42_ and later immunolabelled for Scara1 (red), human Aβ (6E10, green; white arrows indicate intracellular Aβ within vesicles in these phagocytic cells), and nuclei (DAPI, blue). **(C)** Quantitative immunocytochemistry (ICC) analysis of 6E10^+^ IR area (μm^2^/cell #) following intracellular uptake of the 3 Aβ_42_ species (fAβ_42_, pAβ_42_, or oAβ_42_) by MΦ^ACE10^ versus MΦ^WT^ in a period of 15 min. **(D)** Quantitative ICC of cell surface CD36 scavenger receptor expression (μm^2^/cell #) in MΦ^ACE10^ versus MΦ^WT^ following 15-min exposed to the three types of Aβ_42_ species. **(E)** Quantitative analysis of colocalized 6E10^+^ within EEA1^+^ endosomes in MΦ^WT^ versus MΦ^ACE10^ following exposure to the three types of Aβ_42_ species. **(F)** Representative flow cytometry peak analysis in % MFI of HiLyte Fluor 647-labelled fAβ_42_ uptake by MΦ^ACE10^ at the 0-, 5- and 15-min time points. **(G)** MFI analysis of HiLyte Fluor 647-labelled fAβ_42_ uptake by MΦ^ACE10^ at the 0-, 5- and 15-min time points, with and without lisinopril pretreatment. **(H)** MSD biochemical assay determined TNFα levels in MΦ^WT^ versus MΦ^ACE10^, either unstimulated (−) or stimulated (+) with Aβ_42_ fibrils [100 nM] for 24 h. MFI, Median Fluorescence Intensity. Illustration in panel A created with Biorender.com and other panels modified or adapted from (Koronyo-Hamaoui et al., 2020) by permission of Oxford University Press.

**FIGURE 3:**
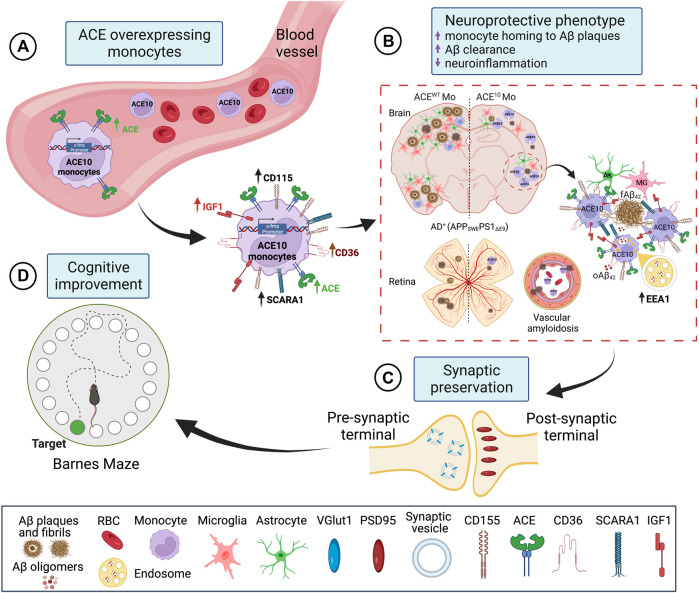
Proposed mechanism for neuroprotective role of ACE overexpression in monocytes in AD. **(A)** ACE overexpression under the activation of the c-fms promotor in CD115^+^ monocytes is named ACE10 model. CD115^+^ ACE10-monocytes exhibit a neuroprotective phenotype with lower expression of pro-inflammatory mediators such as iNOS and TNFα and increased expression of the neurotrophic factor IGF-1, Aβ-degrading enzymes (ACE, MMP-9), and surface scavenger receptors as CD36 and Scara-1 that facilitate phagocytosis of Aβ. **(B)** CD115^+^ ACE10-monocytes are recruited in larger numbers than WT monocytes to AD-like brains and retinas, resulting in neuro-immunomodulation. The ACE10 monocyte-derived macrophages aggregate at areas of amyloid plaque deposition and facilitate the clearance of Aβ oligomers and plaques. This is concomitant with reduced microgliosis and astrogliosis. Notably, in the ACE10 versus WT macrophages, there is increased presence of Aβ assemblies within EEA1 endosomes, suggesting enhanced intracellular lysosomal Aβ degradation machinery in ACE10 macrophages. **(C)** Murine models of AD treated with ACE10 monocytes show reduced AD-related pathology, preservation of excitatory synapses, and **(D)** improved cognitive functions. Created with Biorender.com.

Given the evident reduction in astrogliosis and neuroinflammation, the implications of these for preservation of cognition was explored *in vivo* after adoptive transfer of CD115^+^ monocytes as described above. Barnes Maze tests comparing diseased AD^+^ mice with blood enrichment of either CD115^+^-ACE^10/10^ or CD115^+^-ACE^WT/WT^ monocytes, and healthy WT controls demonstrated preservation of spatial learning and memory capacity in AD^+^ mice following CD115^+^-ACE^10/10^ monocyte adoptive transfer, with virtually no difference compared to the healthy WT mice of similar age (Figure 1M). This was also reflected in immunohistochemical analysis whereby these mice had preservation of pre- and post-synaptic marker densities, far fewer hippocampal and total brain plaques and lower CAA scores compared to diseased controls (Figures 1N,O). These phenotypic changes in ACE^10/10^ monocytes and macrophages were not restricted to cytokine expression or changes in cell morphology. Quantitative immunocytochemistry (ICC) analysis of primary macrophage cultures (Figure 2A) demonstrated a 2-fold increase in the surface binding of soluble oAβ_42_ by ACE^10/10^ macrophages (Figure 2C) with a significant increase in Scara1 (Figure 2B), TREM2, CD163, and CD36 (Figure 2D) surface scavenger expression. In previous studies, these receptors have been associated with increased Aβ uptake (Hickman et al., 2008; Frenkel et al., 2013; Kim et al., 2017). ACE^10/10^ macrophages even demonstrated enhanced endosomal-lysosomal and extracellular degradation of Aβ_42_ (Figure 2E). Furthermore, using the ACE inhibitor lisinopril it was confirmed that ACE catalytic activity is crucial for clearing fAβ_42_. Fibrillar Aβ_42_ uptake was markedly reduced in ACE^10/10^ monocytes pretreated with lisinopril compared to control not treated ACE^10/10^ monocytes (Figure 2G). In addition to highlighting ACE catalytic activity, the *in vitro* analysis further solidified the neuroprotective effects these ACE^10/10^ monocytes have in response to fAβ_42._ The immune profile of these cells was analyzed using sensitive MSD (Meso Scale Discovery) multiplex inflammatory assay. Stimulation of both WT and ACE^10/10^ monocytes with fAβ_42_ resulted in increased expression of TNFα, that was however increased to a much lower extent in ACE^10/10^ monocytes (Figure 2H). This was also true for other important pro-inflammatory cytokines such as IL-6, CXCL1 and IL-1β. This suggests that ACE^10/10^ monocytes, when exposed to Aβ_42_, shifts the inflammatory milieu towards a more neuroprotective state with a less profound increase in destructive inflammatory cytokines when compared to the WT monocytes exposed to the same Aβ_42_ levels. It is possible that the ACE overexpressing monocytes, not only increase expression of this important proteolytic enzyme but also exhibit a distinct anti-inflammatory and neuroprotective phenotype that may prove to be a powerful tool in ameliorating AD pathogenesis. The proposed mechanism for the neuroprotective role of ACE10 monocytes in AD is summarized in Figure 3.

## 5 Conclusion

In recent years histological, biochemical, and genetic studies have demonstrated the association between ACE activity and the development of AD pathophysiology. While the biochemical enzymatic ability of ACE in degrading pathogenic Aβ has been well described, more recent studies demonstrate that ACE overexpression induces a phenotypic change in circulating monocytes and tissue macrophages. The circulating monocytes can home to regions of plaque deposition and possess a unique neuroprotective phenotype with an enhanced ability to phagocytose and clear pathogenic Aβ. This represents an extraordinary opportunity to exploit this natural mechanism of ameliorating AD disease progression. More studies are needed to determine ways in which endogenous ACE can be upregulated in patients suffering from this debilitating illness.
